# High-Grade Endometrial Stromal Sarcoma with *NTRK* Fusion and Response to Larotrectinib: A Case Report

**DOI:** 10.3390/jcm15134887

**Published:** 2026-06-23

**Authors:** Tomer Bar-Noy, Rebecca Lozano-Franco, Teddy S. Nagaria, Melica Nourmoussavi Brodeur, Shannon Salvador, Susie Lau

**Affiliations:** 1 Division of Gynecologic Oncology, Jewish General Hospital, McGill University, Montreal, QC H3T 1E2, Canada; 2 Department of Obstetrics and Gynecology, McGill University, Montreal, QC H4A 3J1, Canada; 3 Department of Pathology, Jewish General Hospital, McGill University, Montreal, QC H3T 1E2, Canada; 4 Lady Davis Institute for Cancer Research, Jewish General Hospital, McGill University, Montreal, QC H3T 1E2, Canada

**Keywords:** larotrectinib, high-grade endometrial stromal sarcoma, treatment, stable disease

## Abstract

**Background**: High-grade endometrial stromal sarcoma (HGESS) is a rare and aggressive uterine mesenchymal tumor with a significant potential for recurrence and metastasis. Advances in molecular pathology have identified recurrent gene fusions involving the neurotrophic tyrosine receptor kinase (*NTRK*) genes, which are crucial for tumorigenesis. The identification of *NTRK* fusions has significant therapeutic implications, as targeted therapies such as Larotrectinib, a selective tyrosine receptor kinase (TRK) inhibitor, have demonstrated remarkable efficacy in *NTRK* fusion-positive tumors across various tumor histologies. **Case Presentation**: This report depicts the case of a 42-year-old woman with HGESS harboring an *NTRK* fusion diagnosed by histopathology and immunohistochemistry after undergoing a vaginal myomectomy. She subsequently underwent a robotic total hysterectomy, bilateral salpingo-oophorectomy, and bilateral lymph node dissection. Following a four-year disease-free interval, HGESS relapsed. The patient received three cycles of gemcitabine plus docetaxel. Subsequent CT imaging indicated progression of the pelvic mass. Molecular testing identified an *NTRK* fusion. Accordingly, larotrectinib was initiated in the setting of progressive disease. After three months, imaging demonstrated a significant decrease in the pelvic mass and near-complete radiographic resolution of the pulmonary nodules. The patient remained on larotrectinib, with January 2024 imaging showing no evidence of recurrence. **Conclusions**: The case presented highlights a personalized approach based on molecular profiling, and the successful use of larotrectinib, a TRK inhibitor, after the identification of an *NTRK* fusion-positive HGESS, emphasizing the importance of molecular diagnostics and targeted therapy in managing this rare malignancy.

## 1. Introduction

High-grade endometrial stromal sarcoma (HGESS) is a rare and aggressive uterine mesenchymal tumor characterized by high mitotic activity and a significant potential for recurrence and metastasis. Recent advances in molecular pathology have identified recurrent gene fusions involving the neurotrophic tyrosine receptor kinase (*NTRK*) genes, which play a crucial role in tumorigenesis. The identification of *NTRK* fusions has significant therapeutic implications, as targeted therapies such as Larotrectinib, a selective tyrosine receptor kinase (TRK) inhibitor, have demonstrated remarkable efficacy in *NTRK* fusion-positive tumors across various tumor histologies. This case report (see Table S1. CARE checklist) discusses the clinical course of a 42-year-old woman with HGESS harboring an *NTRK* fusion, emphasizing the importance of molecular diagnostics and targeted therapy in managing this rare malignancy.

## 2. Case Presentation

A 42-year-old multiparous woman (G7P6A1) presented to the emergency department in March 2017 with intermittent vaginal bleeding lasting several weeks. On physical examination, a prolapsed mass of approximately 5 cm in size was appreciated, initially suggestive of a leiomyoma. A pelvic ultrasound revealed a mildly enlarged uterus containing a 6 mm hypoechoic lesion on the anterior wall and an endometrial thickness of 5 mm. Both ovaries appeared normal, and no other abnormalities were noted.

Given the patient’s clinical presentation and findings, she underwent vaginal myomectomy in March 2017, during which a necrotic 5 cm mass attached to the posterior lip of the cervix was excised. Histopathological analysis revealed a high-grade round and spindle cell sarcoma involving the cervix, exhibiting 12–18 mitoses per 10 high-power fields (HPF). Immunohistochemistry demonstrated positivity for pan-TRK, indicating the presence of an *NTRK* fusion. Additional immunohistochemical markers were negative for epithelial, vascular, muscular, and melanoma markers, supporting the diagnosis of HGESS. Of note, molecular testing was not part of the routine diagnostic *NTRK*workup in 2017, as *NTRK*-rearranged uterine sarcomas had only begun to emerge as a distinct molecular entity. Additionally, routine molecular testing for *NTRK* fusion was not pursued at that time because TRK inhibitors were not yet available as part of routine clinical care.

Subsequently, the patient underwent a robotic total hysterectomy, bilateral salpingo-oophorectomy, and bilateral lymph node dissection. Final pathology confirmed HGESS with extension into the lower uterine segment. The residual tumor measured 5 mm, with no lymphovascular invasion, negative margins, and no lymph node involvement. Cytology from pelvic washing was negative for malignant cells.

Thereafter, the patient declined adjuvant therapy but was monitored closely. Postoperative PET-CT scans in September 2018 and August 2019 showed no evidence of disease. However, in May 2021, after a four-year disease-free interval, she presented with a bowel obstruction and ureteral compression due to a large pelvic mass measuring 13.1 × 11.5 cm. A biopsy confirmed recurrent HGESS, and targeted RNA-based next-generation sequencing (NGS) was performed using the AmpliSeq Focus Fusion Panel (Illumina, Inc., San Diego, CA, USA), which identified an LMNA::*NTRK*1 fusion. No additional gene fusions were detected. The patient required a diverting colostomy and bilateral nephrostomy tube placement due to the mass effect. At this time, the patient agreed to undergo chemotherapy.

## 3. Treatment Response and Complications

### 3.1. Treatment Response

The patient received three cycles of Gemcitabine and Docetaxel chemotherapy until July 2021. A follow-up CT scan demonstrated progression of the pelvic mass, now measuring 15.5 × 14.2 cm, with a new 1.3 cm right lower lobe lung nodule concerning for metastasis. Given the presence of an *NTRK* fusion gene mutation on molecular testing, larotrectinib, an *NTRK* inhibitor, was initiated.

After three months of Larotrectinib treatment, follow-up imaging revealed a significant decrease in the pelvic mass and nearly complete resolution of the lung nodules. A CT scan in May 2022 demonstrated a residual right presacral peri-iliac mass measuring 8.2 × 6.6 cm encasing the internal iliac artery. The patient subsequently underwent secondary debulking surgery, including omentectomy, in July 2022. Due to significant adhesions and the location of the mass, internal iliac artery ligation was required to control bleeding after a laceration of the external iliac vein and internal iliac artery occurred upon lifting the mass. Final pathology revealed degenerative fibro-adipose tissue with no residual malignancy. In November 2022, the stoma reversal was completed with final pathology showing only a benign bowel segment with reactive changes consistent with the colostomy site.

In March 2023, a follow-up PET scan detected a slowly growing 1.6 cm right lower lobe lung nodule with mild FDG uptake (SUV 2.6). The nodule was surgically resected, and pathology confirmed early-stage non-small cell lung cancer (NSCLC). The patient remained on Larotrectinib therapy, and as of October 2024, she showed no signs of HGESS recurrence on imaging studies.

### 3.2. Complications

Although the patient was in continued remission as of January 2024, she nonetheless required medical attention for other events that could be related to her malignancy or ongoing treatment with Larotrectinib. Notably, the patient started experiencing bilateral knee pain since starting Larotrectinib, accompanied by a non-inflammatory right knee effusion. The knee effusion was tapped, and cytology findings were negative for malignancy. In March 2023, the right knee effusion recurred, and after repeat aspiration and cytology, it was deemed to be a mechanical joint effusion with no inflammatory fluid. Then, in July 2023, she returned with bilateral knee pain and effusions, which seemed to respond to intra-articular steroid injections. A right knee MRI showed tricompartmental degenerative changes, which were deemed unusual for her age. She received a referral to orthopedic surgery and, in August 2025, underwent a total right knee arthroplasty, with a post-operative diagnosis of erosive osteoarthritis with severe synovitis.

Additionally, in December 2023, upon presenting with pleuritic chest pain, the patient was found to have bilateral subsegmental pulmonary embolisms and thus started on an oral anticoagulant, which she will likely require for life according to the thrombosis team at our institution.

## 4. Histopathologic Findings

**Figure 1:**
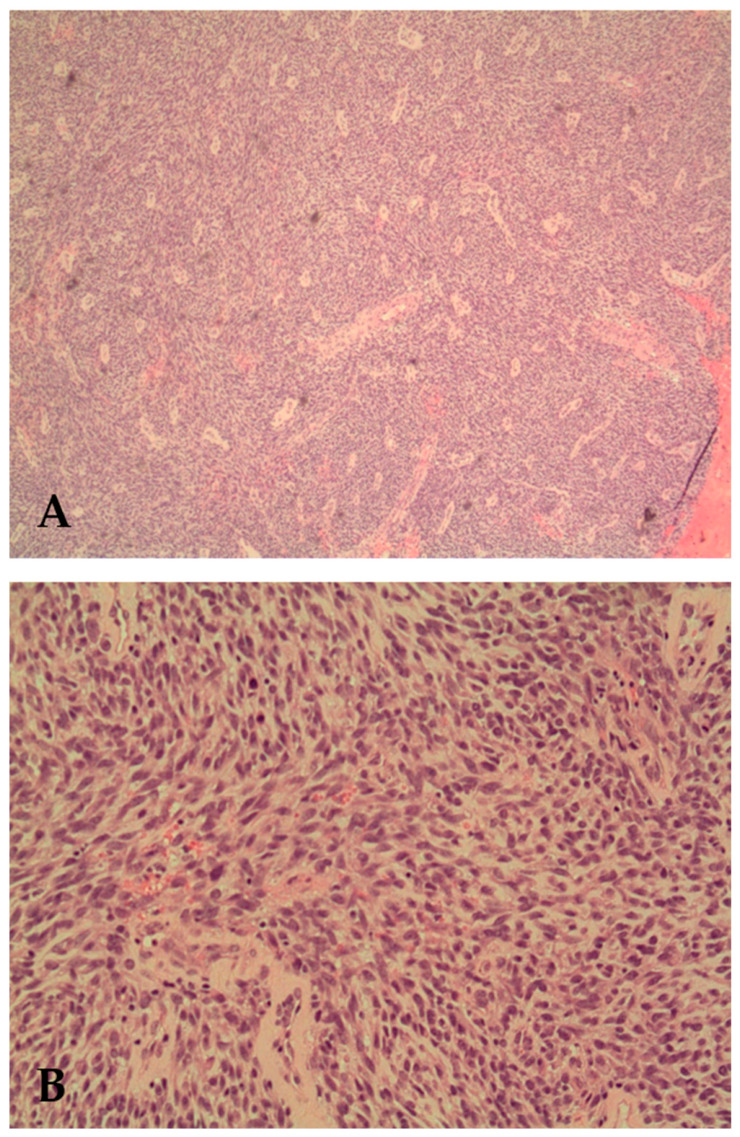
Representative histologic images of high-grade endometrial stromal sarcoma. (**A**) Hypercellular neoplasm with increased vascularity (H&E stain) (40×). (**B**) Neoplasm composed of spindle cells interspersed with epithelioid/round cells with atypia (H&E stain) (200×).

**Figure 2:**
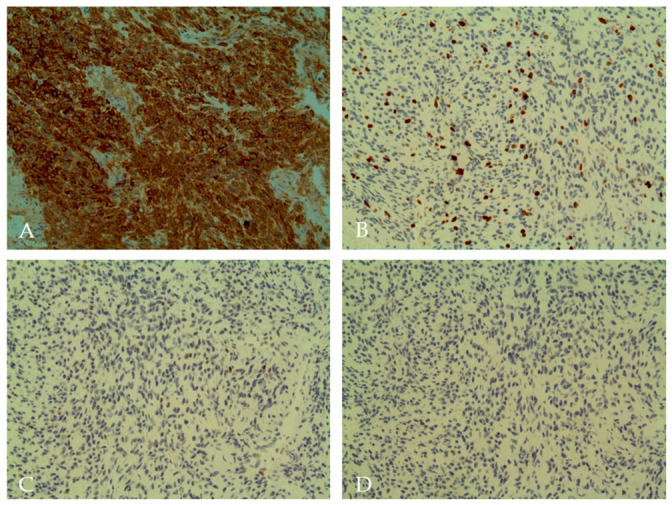
Representative immunohistochemistry (IHC) images of high-grade endometrial stromal sarcoma. (**A**) Neoplastic cells are diffusely positive for pan-TRK immunostain (200×). (**B**) Ki67 proliferative index highlights approximately 25% of the neoplastic cells (200×). (**C**) The p53 stain shows a wild-type pattern (200×). (**D**) Neoplastic cells are negative for pan-keratin immunostain (AE1/AE3 stain) (200×).

**Figure 3:**
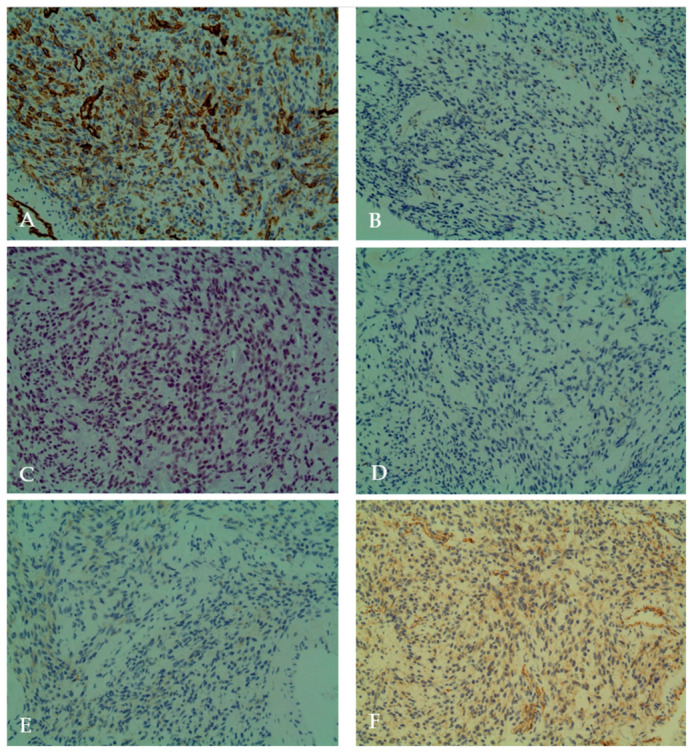
Representative immunohistochemistry (IHC) images of high-grade endometrial stromal sarcoma. (**A**), CD34 highlights neoplastic cells as well as endothelial cells with increased surrounding vessels (200×). (**B**–**D**), Neoplastic cells are negative for cyclin D1, BCOR, and CD10, respectively (200×). (**E**), Neoplastic cells are negative for cKit (200×). (**F**), Neoplastic cells are negative for β-catenin (200×).

Microscopic examination of the tumor (Figure 1) showed a hypercellular proliferation of spindle and round mesenchymal cells with rich capillary-type vascularity infiltrating the cervical stroma. The tumor cells exhibited moderate nuclear atypia and frequent mitoses (14 per 10 high-power fields), including atypical mitotic figures. No tumor necrosis was identified. The residual tumor focus measured 0.5 cm with stromal invasion to a depth of 5 mm. Lymphovascular space invasion was not identified, surgical margins were negative, and both sampled lymph nodes were negative for metastatic disease. The Ki-67 proliferation index ranged from 15% to 25% (Figure 2). Although no validated Ki67 threshold exists for HGESS, increased proliferative activity is generally consistent with the aggressive biological behavior of high-grade uterine sarcomas and should be interpreted in conjunction with morphologic (namely, frequent mitoses and atypical mitotic figures) and molecular findings. Immunohistochemical staining (Figure 2 and Figure 3) was positive for pan-TRK, CD34, CD99 (focal), and STAT6. The tumor cells were negative for a wide panel of markers, including cytokeratins, EMA, CD31, ER, PR, beta-catenin, melanoma markers, muscle markers, cyclin D1, CD45, CD10, BCOR, p16, PAX8, ALK-1, BCL6, WT1, and p63 (Figure 2 and Figure 3). The p53 staining showed a patchy, wild-type pattern (Figure 2). These results support the diagnosis of a HGESS, but lower suspicion for malignancies such as a classic endometrial stromal malignancy (absent CD10), a YWHAE-rearranged HGESS (absence of cyclin D1 expression), a BCOR-driven stromal sarcoma (negative BCOR staining), or alternative mesenchymal tumors (negative c-KIT and beta-catenin). Other defining features, such as biphasic morphology and periglandular stromal hypercellularity, are not present, which renders the diagnosis of adenosarcoma less likely. The absence of cyclin D1, BCOR, CD10, c-KIT, and β-catenin expression, together with diffuse pan-TRK positivity and subsequent molecular confirmation of an *LMNA*::*NTRK*1 fusion, supports the final diagnosis of HGESS with *NTRK* fusion.

## 5. Discussion

Endometrial stromal sarcoma (ESS), although it accounts for less than 1% of uterine malignancies, represents a challenging entity in the realm of uterine malignancies. The 2014 World Health Organization (WHO) classification scheme categorizes ESS into low-grade ESS (LGESS) and high-grade ESS (HGESS), while undifferentiated uterine sarcoma (UUS) remains a separate entity [1]. Traditionally, treatment strategies for ESS have relied on surgical resection, chemotherapy, and radiation therapy. However, the discovery of recurrent gene fusions has revolutionized our understanding of this tumor type and opened new avenues for targeted therapies.

The case hereby reported presents some limitations, notably the delay between positive pan-TRK immunohistochemistry and molecular characterization of *NTRK* fusion, which is attributable to the chronology of events and historical context of the investigations as previously explained. Nonetheless, it highlights the aggressive nature of HGESS and the importance of molecular diagnostics in guiding therapy. In this case, *NTRK* gene fusions have been identified in a subset of uterine sarcomas and are considered driver mutations leading to the constitutive activation of TRK proteins, promoting tumor growth and survival [2,3]. After its original description in 2018 [2], a consistently increasing number of *NTRK* gene fusion sarcomas are being described in the literature [4,5]. Table 1 provides a comparison of various cases of *NTRK* gene fusions in uterine sarcomas described in the literature.

Larotrectinib, a highly selective TRK inhibitor, has shown efficacy across a variety of *NTRK* fusion-positive tumors. In pre-clinical models, blocking TRK signaling has been shown to decrease tumor growth and to increase apoptosis in *NTRK* fusion-positive tumor cells [6]. In clinical trials, larotrectinib has demonstrated substantial benefits: in a study involving a broad range of tumor types, 79% of patients with a TRK fusion-positive cancer treated with larotrectinib had an objective response, with 16% having a complete response, including a subset with sarcomas [7]. Similarly, Demetri et al. report a 74% larotrectinib objective response rate (ORR) in the adult population with *NTRK* fusion-positive sarcomas, while the ORR in the pediatric population is as high as 94% [8]. This data emphasizes the robust clinical benefits achieved from TRK inhibition, particularly in sarcoma patients. Furthermore, clinical trials have shown promising results in other tumor types, such as neuroblastoma, infantile fibrosarcoma, secretory breast carcinoma, salivary gland carcinoma, thyroid carcinoma, and lung cancer [7,8,9]. In our case, the patient experienced significant tumor regression and prolonged disease control with larotrectinib therapy, which is consistent with the reported outcomes.

While pan-TRK immunohistochemistry is a valuable screening tool that exhibits high sensitivity (75–100%) and specificity (93–100%) for detecting *NTRK* rearrangements, it is not a definitive diagnostic tool [10]. Pan-TRK immunohistochemistry positivity should thus be interpreted cautiously; Moura et al. investigated the potential pitfalls of pan-TRK immunohistochemistry in gynecological mesenchymal tumors and found that an accurate diagnosis requires confirmation through molecular methods such as next-generation sequencing (NGS) or fluorescence in situ hybridization (FISH) to determine the specific fusion pattern [10].

Moreover, the genetic landscape of uterine sarcomas is undeniably complex and rapidly evolving. Indeed, it has been significantly redefined by the discovery of recurrent gene fusions [3]. These molecularly defined subtypes, including those harboring *NTRK* fusions, highlight the importance of comprehensive molecular profiling and personalized therapy in managing these rare and complex malignancies. These tumors were previously categorized as UUS, but with the advent of molecular profiling, they are now recognized and addressed as distinct entities with specific molecular and clinical features. This underscores the importance of pathologists being aware of these emerging categories to be able to consider molecular testing in cases of uterine sarcomas with ambiguous histopathological features. Indeed, several genetic rearrangements can occur, including those involving *NTRK*, YWHAE-NUTM2A/B, and BCOR genes [1,3]. These rearrangements can result in a wide range of histological presentations, which is essential information for accurate diagnosis and targeted therapy for different tumor subtypes [1,3].

Recent guidelines emphasize the importance of molecular testing for *NTRK* fusions in uterine sarcomas, particularly for high-grade tumors. The French Sarcoma Group and Rare Gynaecological Tumors recommend screening for these fusions, given their significant therapeutic implications [11]. In general, sarcomas, including HGESS, often present a challenge in management due to their heterogeneity and varied response to standard chemotherapeutic agents. While chemotherapy is a common first-line treatment for many sarcomas, the response rates can be inconsistent depending on the histological subtype [12].

For patients with *NTRK* fusion-positive tumors, targeted therapy has shown substantial promise. The emergence of Larotrectinib provides a compelling case for molecular testing in sarcomas, as highlighted by Drilon et al., who reported significant response rates in cases with such fusions [9]. Other agents, such as trabectedin, have been shown to benefit patients with advanced uterine leiomyosarcomas, often used as a maintenance agent or in combination with doxorubicin, with one case report highlighting the achievement of stable disease in HGESS with trabectedin combined with radiotherapy [13,14]. Indeed, trabectedin is a substance derived from a type of marine invertebrate that is currently used in previously treated advanced soft-tissue sarcoma. In addition, several studies have suggested a potential role for immunotherapy in the treatment of sarcomas, particularly with the use of immune checkpoint inhibitors, natural-killer cell-based immunotherapy, and bifunctional proteins that target both TGF-β and PD-L1 [15,16,17]. This approach, which aims to stimulate the immune system to fight cancer cells, holds promise for patients with advanced or recurrent disease.

## 6. Conclusions

In reviewing the management of HGESS, it is essential to consider the range of treatment options available, particularly in the context of the patient’s clinical course. In recent years, the landscape of uterine sarcomas has been redefined by the discovery of recurrent gene fusions. These molecularly defined subtypes, including *NTRK*, YWHAE, and BCOR fusion-positive tumors, highlight the importance of comprehensive molecular profiling and personalized therapy in managing these rare but complex malignancies. The use of NGS and other advanced molecular technologies has revolutionized the diagnosis and treatment of uterine sarcomas, offering new hope for patients with these often aggressive tumors. The case presented highlights a personalized approach based on molecular profiling and the successful use of larotrectinib, a TRK inhibitor, after the identification of an *NTRK* fusion-positive HGESS.

Continued research on *NTRK* fusion-positive tumors will be key to further establishing effective treatment protocols and improving patient outcomes in this rare and aggressive neoplasm.

## Figures and Tables

**Table 1:** Comparison table of reported cases of uterine sarcomas with *NTRK* gene fusions.

Feature	Chiang et al. (2018) [2]	Boyle et al. (2020) [4]	Costigan et al. (2023) [5]
**Number of cases**	4	1	15
**Age (years)**	Median 44 (27–47)	42	Median 35 (16–61)
**Location**	Cervix (*n* = 3) Uterus corpus (*n* = 1)	cervix	Cervix (*n* = 14) Uterus corpus (*n* = 1)
**Stage at diagnosis**	FIGO stage IB (*n* = 4)	Uterus-confined	Organ-confined (*n* = 13)
**Histopathologic** **description**	Polypoid or intramural masses, fascicular fibrosarcoma-like spindle cell proliferation, high mitotic activity (7–30/10 HPF), necrosis (*n* = 2), no lymphovascular invasion	Polypoid cervical lesion, dense short fascicles of monomorphic spindle cells, high mitotic activity, absence of necrosis, no lymphovascular invasion reported	Cervical spindle-cell sarcomas with infiltrative growth, infiltrative fascicular spindle-cell neoplasms, high mitotic activity ^a^ and necrosis, some cases with lymphovascular invasion ^b^
**Pan-TRK result**	Positive (*n* = 4)	Positive	Positive in all tested individuals (*n* = 13)
***NTRK* fusion patterns**	*TPM3*-*NTRK*1, *LMNA*-*NTRK*1, *TPR*-*NTRK*1, *RBPMS*-*NTRK*3	*TPM3*-*NTRK*1	*NTRK*1 fusions (*TPR, TPM3, EML4, TFG, SPECC1L, C16orf72*) and *NTRK*3 fusions (*IRF2BP2* and more)
**Molecular testing**	RNA sequencing, FISH	FISH, NGS	RNA and DNA sequencing
**Outcomes**	Recurrence or metastasis (*n* = 2), death (*n* = 1)	No recurrence	Death (*n* = 3) ^c^

^a^ Mitotic activity > 8/10 HPF was an adverse prognostic factor. ^b^ Necrosis and lymphovascular invasion were associated with worse outcomes. ^c^ Among 9 patients who received follow-up.

## Data Availability

The original contributions presented in this study are included in the article. Further inquiries can be directed to the corresponding author.

## Supplementary Materials

The following supporting information can be downloaded at: https://www.mdpi.com/article/10.3390/jcm15134887/s1, Table S1: CARE checklist.

## Abbreviations

The following abbreviations are used in this manuscript:

ESS	Endometrial stromal sarcoma
FISH	fluorescence in situ hybridization
HGESS	High-grade endometrial stromal sarcoma
HPF	high-power fields
LGESS	low-grade endometrial stromal sarcoma
NGS	next-generation sequencing
NSCLC	non-small cell lung cancer
*NTRK*	neurotrophic tyrosine receptor kinase
TRK	tyrosine receptor kinase
USS	undifferentiated uterine sarcoma
WHO	World Health Organization
